# Eltrombopag for the Treatment of Allogeneic Hematopoietic Stem Cell Transplantation-Related Poor Graft Function

**DOI:** 10.7759/cureus.44555

**Published:** 2023-09-02

**Authors:** Eren Arslan Davulcu, Nur Akad Soyer, Filiz Vural

**Affiliations:** 1 Department of Hematology, University of Health Sciences, Bakirkoy Dr. Sadi Konuk Training and Research Hospital, Istanbul, TUR; 2 Department of Hematology, Faculty of Medicine, Ege University, Izmir, TUR

**Keywords:** stem cell transplantation, allogeneic stem cell transplant (allo-sct), neutropenia, anemia, thrombocytopenia, engraftment, eltrombopag, poor graft function

## Abstract

Introduction: Allogeneic stem cell transplantation (ASCT) is a crucial therapeutic strategy for hematological and non-hematological disorders. Poor graft function (PGF) after ASCT presents a critical challenge that does not have a standardized treatment approach. A thrombopoietin-mimetic oral drug eltrombopag shows promise in some bone failure syndromes. This study aimed to analyze the efficacy of eltrombopag in treating PGF after ASCT.

Methods: Patients receiving eltrombopag for PGF after ASCT between 2017 and 2020 were retrospectively evaluated. Patients’ characteristics, details for ASCT, timing, treatment, and possible contributors for PGF, response to eltrombopag treatment, and overall response rate (ORR) were analyzed.

Results: Eighteen patients were assessed. Eltrombopag treatment yielded a favorable response in 11 patients, resulting in an ORR of 61%. The ORR in secondary PGF was better than that in primary PGF (83% and 17% respectively). There was a marked enhancement in platelet and hemoglobin levels following eltrombopag treatment (*p=0.001* and *p=0.030*, respectively), while neutrophil values exhibited no significant change (*p=0.8*). Among the responding patients, four individuals (22%) underwent a tapering and discontinuation of eltrombopag. No toxicity was observed above grade one, and no patient discontinued eltrombopag because of intolerability or adverse events.

Conclusion: Our findings affirm that eltrombopag can treat poor graft function after allogeneic stem cell transplantation without significant toxicities. These results contribute to the growing body of evidence supporting the use of eltrombopag in poor graft function after allogeneic stem cell transplantation, providing insights into its potential benefits and limitations.

## Introduction

Allogeneic stem cell transplantation (ASCT) is the curative treatment modality for mainly malignant and some non-malignant hematological and non-hematological diseases. After a conditioning regimen (chemotherapy±radiotherapy), patients encounter an aplastic phase. Over time, donor CD34-positive stem cells that have been infused begin to engraft. Eventually, normal blood count replaces the aplastic phase. Engraftment according to cell groups is defined as the first of three consecutive days with an absolute neutrophil count higher than 0.5 × 10^9^/L, sustained >20 × 10^9^/L platelets, and hemoglobin >8 g/dL, free of transfusion requirements [1]. Engraftment of the stem cells usually takes around 10 to 20 days, depending on the type of stem cells used, CD34 cell count, conditioning regimen, accompanying infections, immunosuppression, age, and medications. Lack of engraftment can be seen due to primary and secondary engraftment failure, poor graft function (PGF), and graft rejection [1]. In the presence of complete donor chimerism and without disease relapse, persistent cytopenia is defined as PGF. Cytopenia may be present in one or more cell lineages. PGF which is the subject of our study can be a life-threatening complication.

Treatment of PGF usually remains insufficient because it is usually not possible to find out the underlying problem. Management strategies are growth factor treatment [2], mesenchymal stem cell transfusion [3], CD34-positive stem cell boost [4], and second ASCT [5]. Many of these treatments are difficult approaches that are not suitable for every patient. A thrombopoietin-mimetic oral drug eltrombopag has been successfully used for the treatment of immune thrombocytopenic purpura, aplastic anemia, and some myelodysplastic syndrome-related thrombocytopenias [6]. In recent years, data on the use of eltrombopag in the treatment of PGF have been published [7-10]. Improvement with eltrombopag was observed not only in platelets but also in all three cell lines. This study aimed to analyze the single-center experience of eltrombopag use in post-ASCT PGF.

## Materials and methods

Patient population

Between August 2017 and September 2020, patients over 18 years old, who underwent ASCT for malignant and non-malignant diseases, were retrospectively evaluated. Those patients who received eltrombopag for PGF after ASCT were analyzed. The study was approved by the local ethics committee (Ege University Ethical Committee, date 15.11.2020, number 20-11T/80). Informed consent was obtained from all patients or their first-degree relatives before starting the study. 

Diagnosis of PGF

PGF was defined as the presence of persistent thrombocytopenia (<20× 10^9^/L) at >35 days from ASCT, with or without other cytopenias, with full donor chimerism. According to the initial normalization of platelet count, PGF was divided into primary and secondary. Primary PGF was defined as a requirement for platelet transfusion because of insufficient platelet recovery (<20× 10^9^/L), and secondary PGF was defined as insufficient platelet recovery (<20× 10^9^/L) occurring after the initial engraftment (platelets >50× 10^9^/L) without transfusions and in the absence of relapse of the original disease. The involvement of erythroid and myeloid lineages was defined as persistent anemia (hemoglobin < 9.0 g/dL), and persistent absolute neutrophil count (ANC) <1.0 × 10^9^/L.

In addition to the blood count, a peripheral blood smear was performed to check platelet numbers. Bone marrow aspiration and biopsy, abdominal ultrasound and portal Doppler ultrasound, biochemistry examination including transaminases, chimerism tests, and cytomegalovirus (CMV) DNA count were performed for differential diagnosis. The status of the primary disease, bone marrow fibrosis, and megakaryocyte count was examined in bone marrow biopsy. Bone marrow fibrosis is graded in four levels, from grade zero to grade three.

To investigate underlying causes for PGF, the co-existence of graft versus host disease (GVHD), CMV infection/reactivation, other infections, and drug use (chemotherapy, antibiotics, etc.) were evaluated. It was categorized as ‘idiopathic PGF’ if there was no underlying cause. The hematopoietic cell transplantation-comorbidity index (HCT-CI) scores [11] and donor type were also recorded.

Eltrombopag treatment

Eltrombopag treatment was initiated between 25 and 50 mg, increasing the dose by 25 mg every two to four weeks up to 150-300 mg/day orally according to the hematological response, side effects, and manufacturer's recommendations. When the hematological response was achieved, the dose was tapered and finally discontinued. Side effects related to eltrombopag were graded according to the National Cancer Institute Common Toxicity Criteria version 4.0 [12].

For clinically stable patients the platelet transfusions threshold was less than 10×10^9^/L. In case of bleeding, invasive procedures, fever, or anticoagulant therapy, patients were transfused at higher platelet values.

Hematological response

The complete response (CR) was defined as platelet ≥ 50 × 10^9^/L, neutrophil ≥ 1.0 × 10^9^/L, and hemoglobin ≥ 9 g/dL, without blood cell transfusion or granulocyte colony-stimulating factor for ≥ 7 consecutive days. The partial response (PR) was defined as hematopoietic engraftment of at least two lineages (platelet ≥ 20 × 10^9^/L, neutrophil ≥ 0.5 × 10^9^/L, and hemoglobin ≥7 g/dL) [13].

Endpoints

The endpoint was the overall response rate (ORR), which consisted of the CR and PR.

Statistical analysis

Statistical analyses were performed using IBM SPSS Statistics for Windows, Version 26 (Released 2019; IBM Corp., Armonk, New York, United States). The characteristics of the patients were presented as median (range) for continuous variables and frequencies (percentages) for categorical variables. Groups were compared using Pearson chi-square tests or Fisher’s exact test for categorical variables and Mann-Whitney U tests for continuous variables. *p* values *<0.05* were considered statistically significant.

## Results

Eighteen patients with PGF after ASCT were evaluated. The median age at diagnosis was 45.5 years (range 23-66 years). Patients’ characteristics are summarized in Table 1.

**Table 1:** Patients' characteristics ALL: Acute lymphoblastic leukemia; AML: Acute myeloblastic leukemia; HCT-CI: Hematopoietic cell transplantation-comorbidity index; HLA: Human leukocyte antigen; MDS: Myelodysplastic syndrome; MF: Myelofibrosis; PNH: Paroxysmal nocturnal hemoglobinuria

Variables		Results
Gender, n (%)	Male	11 (61%)
	Female	7 (39%)
Disease, n (%)	AML	6 (33%)
	ALL	6 (33%)
	MDS	3 (17%)
	MF	2 (11%)
	PNH	1 (6%)
HCT-CI, score, %	0	15 (83%)
	1	1 (6%)
	2	2 (11%)
Donor type, n (%)	HLA-matched related	9 (50%)
	HLA-matched unrelated	8 (44%)
	Haploidentical	1 (6%)
Conditioning regimen, n (%)	Myeloablative	11 (61%)
	Reduced intensity	7 (39%)
Initial bone marrow fibrosis, grade, n (%) (available for 17 patients)	0	2 (12%)
	1	11 (65%)
	2	3 (17%)
	3	1 (6%)
Initial bone marrow megakaryocytes, n (%)	Adequate	9 (50%)
	Decreased	9 (50%)
Baseline laboratory findings	Platelet x 10^9^/L, median (range)	27.5 (7-37)
	Hemoglobin x g/dL, median (range)	9.73 (5-11,46)
	Neutrophil x 10^9^/L, median (range)	3.3 (0.5-4.9)

In all patients, the source of stem cells was peripheral blood. The median CD34-positive cell count infused was 5.25X 10^6^ (range 3.8-11.82 x 10^6^). For GVHD prophylaxis, most of our patients (16 patients, 89%) received standard methotrexate and cyclosporine treatment, and two patients had also post-transplant cyclophosphamide. 

Of seven patients whose both donor/recipient CMV status were available, there were no unfavorable combinations (donor negative/recipient positive). Fourteen recipients were CMV IgG positive, and one recipient was negative. Four donor/recipient pairs (22.2%) showed a major ABO incompatibility.

PGF was primary in six cases (33%) and secondary in 12 cases (67%). In five patients, single lineage cytopenia (only platelets) was detected, in four patients, bilineage cytopenia (in three patients platelets and erythrocytes, in one patient platelets and neutrophil respectively) was detected and, in nine patients, trilineage cytopenia (platelets, erythrocytes, and leukocytes) was detected. PGF was idiopathic in three patients. In the remaining 15 patients, infections and GVHD were accompanied at the specified rates (Table 2).

**Table 2:** Etiology of PFG CMV: Cytomegalovirus; GVHD: Graft versus host disease; PGF: Poor graft function

Variables	n (%)
GVHD	3 (17%)
CMV infection	1 (5,5%)
Idiopathic	3 (17%)
GVHD+ CMV infection	10 (55%)
GVHD+ CMV infection+BK virus infection	1 (5,5%)

Eleven patients responded to eltrombopag treatment (ORR 61%), of those 10 had CR and one PR. Seven patients (39%) were non-responsive. The ORR in secondary PGF was better than that in primary PGF (83% and 17% respectively). A statistically significant increase in platelet and hemoglobin values was observed after eltrombopag treatment (*p=0.001* and *p=0.030* respectively) but not for neutrophil values (*p=0.8*). The median time from ASCT to eltrombopag treatment was 79.5 days (range 59-358 days). The median time to respond for responder 11 patients was 35 days (range 14-58 days). Eltrombopag was tapered and discontinued in four of the responding patients (22%). All patients maintained their response at their last visit (10 patients CR and one PR) and remained transfusion independent at a median of 121 (range 25-721) days. At the last follow-up, 11 patients were alive, seven of whom were still on eltrombopag therapy.

A total of seven patients died at the end of the study period, one due to disease relapse, five due to GVHD and sepsis, and one due to fungal pneumonia. Two patients had grade one anorexia, two patients had grade one fatigue, and one patient had grade one aspartate aminotransferase, alanine aminotransferase, and alkaline phosphatase elevation. However, no patient discontinued eltrombopag because of intolerability or adverse events. Patients’ characteristics, HSCT and PGF details, response to eltrombopag, and outcome are detailed in Table 3. 

**Table 3:** Patients’ characteristics, HSCT and PGF details, response to eltrombopag, and outcome AC: Accompanying condition; aGVHD: Acute GVHD; ALL: Acute lymphoblastic leukemia; AML: Acute myeloblastic leukemia; ANC: Absolute neutrophil count; ASCT: Allogeneic stem cell transplantation; BR: Best response; cGVHD: Chronic GVHD; CR: Complete remission; F: Female; GVHD: Graft versus host disease; Hb: Hemoglobin; M: Male; MDS: Myelodysplastic syndrome; MF: Myelofibrosis; NR: Non-responsive; P: Primary; PGF: Poor graft function; PN: Patient no; PNH: paroxysmal nocturnal hemoglobinuria; PLT: Platelet; RBC: Red blood cell; PR: Partial remission; S: Secondary

PN	Age	Gender	Diagnosis	CD34+ cells infused (x10^6^)	PGF type	AC	Lineage involvement	Hb (g/dL) (Pre-/posttreatment)	PLT(10^9^/L) (Pre-/posttreatment)	ANC(10^9^/L) (Pre-/posttreatment)	Time to response (days)	BR	Outcome
1	66	M	MF	4.8	S	CMV, cGVHD	PLT	9.4/8.92	18/99	1.8/2.2	35	CR	Exitus
2	59	M	MF	6	S	cGVHD	PLT, RBC	7.08/10.3	12/84	1.4/4.6	39	CR	Alive
3	66	F	AML	6.17	P	CMV	PLT, RBC	8.86/7.12	20/19	0.9/0.9	-	NR	Exitus
4	23	M	AML	5	P	-	PLT, RBC, ANC	6.35/7.4	13/16	0.72/0.75	-	NR	Exitus
5	37	M	AML	6.36	P	CMV, aGVHD	PLT, RBC, ANC	6.2/7.8	18/7	0.65/0.3	-	NR	Exitus
6	47	F	ALL	5.08	P	CMV, aGVHD	PLT, RCB, ANC	5.74/7.42	14/23	0.53/0.35	-	NR	Exitus
7	39	M	ALL	9	S	CMV, cGVHD	PLT	8.31/10.1	20/82	3.9/2.7	49	CR	Alive
8	32	M	ALL	11. 82	S	CMV, cGVHD	PLT, RBC, ANC	7.56/9.71	15/127	0.67/1.7	39	CR	Alive
9	53	F	AML	4.4	S	-	PLT, RBC, ANC	6.7/9.3	28/62	0.70/0.74	15	CR	Alive
10	45	F	MDS	3.8	S	-	PLT, RBC, ANC	5/10.6	9/173	0.98/2.2	58	CR	Alive
11	53	M	PNH	5.9	S	CMV, aGVHD	PLT, ANC	9.5/11.9	6/54	0.60/1.2	15	CR	Alive
12	64	M	ALL	5.25	P	CMV, cGVHD	PLT, RBC, ANC	5.7/9.6	21/64	0.37/0.7	48	CR	Exitus
13	60	M	MDS	4.33	S	CMV, cGVHD	PLT, RBC, ANC	5.5/8.6	7/18	0.25/0.60	-	NR	Alive
14	29	F	MDS	6.4	S	CMV, aGVHD	PLT, RBC, ANC	8.14/8	31/109	0.49/1.0	26	CR	Alive
15	48	M	AML	5.11	P	CMV, cGVHD, BK virus	PLT, RBC	8.6/7.2	19/14	4.1/0.28	-	NR	Exitus
16	58	F	ALL	6.73	S	cGVHD	PLT	11.46/10.4	30/150	3.1/3.2	35	CR	Alive
17	34	M	ALL	5.9	S	CMV, aGVHD	PLT	12.2/12.8	23/40	3.1/1.7	14	PR	Alive
18	34	F	AML	5.71	S	aGVHD	PLT	10.06/7.2	3/21	4.9/0.65	-	NR	Alive

## Discussion

Eltrombopag has long been used successfully in immune thrombocytopenic purpura and aplastic anemia. In recent years, retrospective data on its use for post-HSCT PGF have been increasing. Although it is not known exactly how eltrombopag may be effective in bone marrow failure syndromes of various etiologies, there are many assumptions. It is thought that eltrombopag affects all CD34-positive stem cells via c-Mpl for maturation; furthermore, it promotes immunomodulation [14]. Eltrombopag is a thrombopoietin-mimetic, but it improves not only thrombocytopenia but also cytopenias of other series. Although definitions for PGF do not show homogeneity among studies, cut-off values for cytopenias are close to each other.

The study by Tang et al. included only secondary PGF, patient characteristics were similar, but response rates were higher compared to our study (ORR were 83.3% vs. 61% respectively). The time from eltrombopag initiation to achieving response was similar in both groups (29 vs. 35 days) [15]. Although the details in the other study are not known, the relatively low response rate in our study was thought to be due to the presence of severe 'secondary' PGF causes.

The study by Marotta et al. is very similar to this study in terms of PGF definition and patient characteristics [9], but the source of stem cells in this study was bone marrow and the amount of CD34-positive stem cells infused into patients was lower than ours (2.24 vs 5.25 × 10^6^ /kg). The ORR and time-to-response times were found to be similar in both studies. In addition, this study investigated whether the hematological response was affected by the putative cause of PGF. They found that the hematological response to eltrombopag was seen in 3/5 patients (60%) with a putative immune cause, in 5/8 (62%) of patients with a putative infectious/iatrogenic cause, and 1/3 (33%) of patients in whom pathogenic causes remained elusive. It has been emphasized that the improvement in PGF is not dependent on eltrombopag alone but may be related to the improvement of these underlying causes [9]. As the cause of PGF, we would like to emphasize that these secondary factors often co-exist and they have an intricate effect both in the formation of the disease and in the healing process.

A more comprehensive, multi-center study by Giammarco et al. investigated the predictors for response to eltrombopag treatment for PGF following allogeneic stem cell transplant. Positive predictors of response were an HLA-matched donor, a CD34-positive dose at transplant >4× 10^6^/kg, and starting eltrombopag treatment at least 90 days after HSCT [13].

A single-center but comprehensive study by Kırcalı et al. also included autologous stem cell transplantation-related PGF and found that platelet, neutrophil, and hemoglobin counts increased statistically significantly after eltrombopag treatment compared to pretreatment. It is important that 61.5% of the patients were able to discontinue eltrombopag successfully after a median time of 82 days, significantly better than our discontinuation rate (22%) [16].

This study has limitations regarding its limited patient number and retrospective nature, as it was conducted in a single center. The fact that the definition of PGF is not clear and there are differences between studies can cause problems in comparing studies with each other. The multi-factorial nature of PGF and their contributions to the pathogenesis and healing process of the underlying diseases create a complex effect.

## Conclusions

This single-center study investigated real-world data on allogeneic stem cell transplantation-related poor graft function. The complex and multi-factorial nature of the cytopenias after allogeneic stem cell transplantation necessitates a comprehensive approach that includes patient characteristics, underlying causes, and treatment strategies. Our study supported that eltrombopag is successful in the treatment of poor graft function without major toxicities. Subsequent multicenter studies with larger cohorts and the establishment of standardized poor graft function definitions will help enhance treatment recommendations and provide insights into factors affecting response variability.
